# Differences Between the 2016 and 2022 Editions of the Enhanced Recovery After Bariatric Surgery (ERABS) Guidelines: Call to Action of FAIR Data and the Creation of a Global Consortium of Bariatric Care and Research

**DOI:** 10.1007/s11695-022-06132-7

**Published:** 2022-06-02

**Authors:** Bart Torensma, Mohamed Hisham, Abdelazeem A. Eldawlatly, Mohamed Hany

**Affiliations:** 1 Clinical Epidemiologist, Leiden University Medical Center (LUMC), Albinusdreef 2, 2333 ZA Leiden, The Netherlands; 2 Medical Research Institute, Alexandria University, Alexandria, Egypt; 3 Head of the Department of Anaesthesiology, King Saudi University Hospital, Riyadh, Saudi Arabia; 4 Head of the Department of Surgery, Medical Research Institute, Alexandria University, Alexandria, Egypt

**Keywords:** ERAS, ERABS, FAIR, Bariatric surgery

## Abstract

In 2016, the Enhanced Recovery After Bariatric Surgery guidelines (G16) was published, and in 2022, an update to it was released (G22). Grading of recommendations, assessment, development, and evaluations (GRADE), emphasizing the level of evidence (LoE) of both the guidelines, was performed. An overview of methodology was also performed, considering the following questions: how can research be improved, what can be done in the future using data, and how to collaborate more? Both guidelines did not explain how the LoE conclusions were derived regarding the risk of bias. There is also potential for forming a global consortium that deals with bariatric research, which can serve as a repository for all relevant data. Ensuring that this data is FAIR (findability, accessibility, interoperability, reusability) compliant and using this data to formulate future guidelines will benefit clinicians and patients alike.

## Introduction

The Enhanced Recovery After Surgery (ERAS) is a model of care introduced in 1997 by a group of general surgeons from Northern Europe led by Henrik Kehlet [1–3]. The core tenet of this approach was to improve patient outcomes following surgery, especially in terms of hospital stay, complications rate, early recovery, and reduction of economic burdens.

Since bariatric surgery can be very protocolized, it was necessary to create one to enhance recovery after bariatric surgery. Therefore, in 2016, the first such protocol was introduced by Thorell et al. [4], the so-called Enhanced Recovery After Bariatric Surgery (ERABS). It focused on all the aspects around the procedure itself and patients with obesity in terms of safety and outcome. In 2022, Stenberg et al. [5] introduced a 2021 update version of this same protocol. Both protocols used extensive literature sourced from all known databases PubMed, EMBASE, and Cochrane databases [6] and ClinicalTrials.gov and the Grading of Recommendations, Assessment, Development and Evaluation (GRADE) system [7–9]. GRADE is the gold standard for evaluating the quality of research.

Grading is classified from high, wherein the actual effect lies close to the estimate of the impact, to very low, wherein the real impact is likely to be substantially different from the assessment of the effect. Both protocols describe all the grading well, but they do not mention what criteria were used to arrive at the grading score and how further research has changed or altered specific aspects of care over 6 years.

This review describes all the observations between 2016 and 2022 on how the score changed, the risk of bias assessment findings, and how guided, collaborative research can be beneficial to the next update.

## Method

This review checked two guidelines regarding perioperative care in bariatric surgery and described the changes over time in both guidelines (ERABS): from the ERABS guideline in 2016 (G16) (first edition) [4] and the ERABS guideline in 2022 (G22) (a 2021 update, second edition) [5].

Depending on the results, a recommendation is given for the future, based on the latest methodology, research, and statistics insights.

Two reviewers (Torensma and Hisham) independently screened all elements, basis of recommendations, and level of evidence (LoE) (as per GRADE) in this review. Disagreements were solved via discussion or by consulting a third independent reviewer (Hany). G16 involved a review of the literature published between January 1966 and January 2015. G22 included literature published till 2020.

The scoring of each element was investigated, on how it was assigned an LoE and how it impacted recommendations, and then results were compared. This gave three possible results when comparing G16 and G22 results: “same,” “increased,” or “decreased.” All the elements that contained sub-elements were scored separately. The results of this review include both the primary and sub-elements together.

A methodology overview was also performed on how research can be improved, facilitating GRADE concerning results for each element. The questions of what can be done in the future regarding guideline formulation using FAIR data and how research can be more collaborative at a global level were also analyzed and discussed under the purview of this review.

## Results

### Search Strategy

Both G16 and G22 did a comprehensive search and used appropriate keywords and medical subheading (MESH) terms. The databases surveyed and the terms used include:

#### G16

PubMed, EMBASE, and Cochrane databases and ClinicalTrials.gov through December 2015.

Keywords included “obesity,” “morbid obesity,” “bariatric surgery,” “metabolic surgery,” “gastric bypass,” “sleeve gastrectomy,” one anastomosis gastric bypass,” “mini-gastric bypass,” “gastric banding,” “fast track,” and “enhanced recovery.”

#### G22

PubMed, EMBASE, and Cochrane databases and ClinicalTrials.gov through December 2020.

Keywords included “obesity,” “morbid obesity,” “bariatric surgery,” “metabolic surgery,” “gastric bypass,” “sleeve gastrectomy,” one anastomosis gastric bypass,” “mini-gastric bypass,” “gastric banding,” “fast track,” and “enhanced recovery.”

### Study Selection

Both G16 and G22 performed a title and abstract screening with individual authors blinded to each other. A third author resolved any disagreement.

### Quality Assessment and Data Analysis

The quality assessment was done appropriately and according to the scientific community’s methodology and advice. Cochrane checklist [6] and GRADE [7–9] were used to guide the process.

All authors determined the strength of each recommendation, and if there was disagreement regarding the power, the Delphi method was used to reach a consensus between all the authors.

The criteria for rating the strength of recommendations were as follows: “Strong recommendation”: There was confidence that the desirable effects of adherence to the recommendation outweigh the undesirable effects. “Weak recommendation”: the desirable results of commitment to the recommendation probably outweigh the unwanted effects, but the panel is less confident.

### Differences Between G16 and G22 Over Time (Table 1)

**Table 1:**
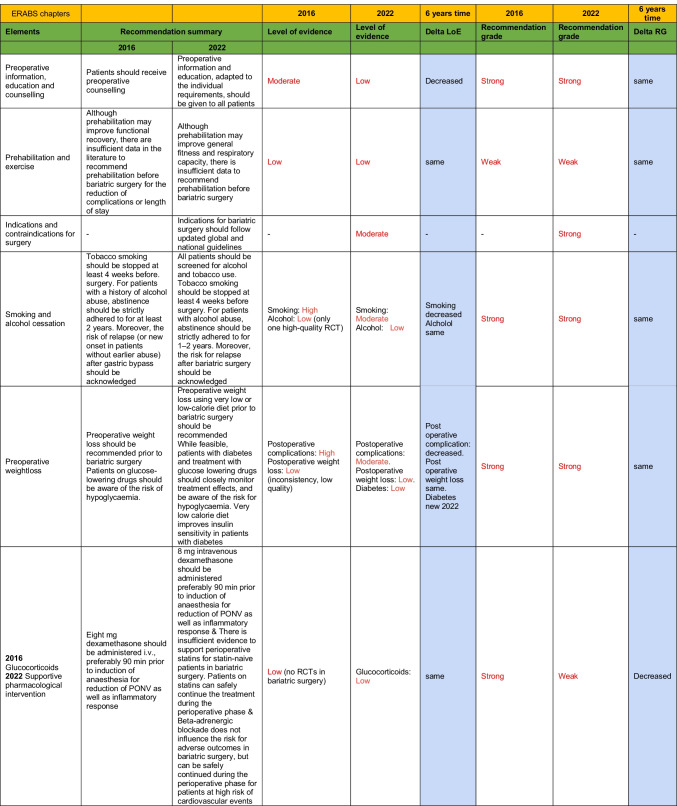

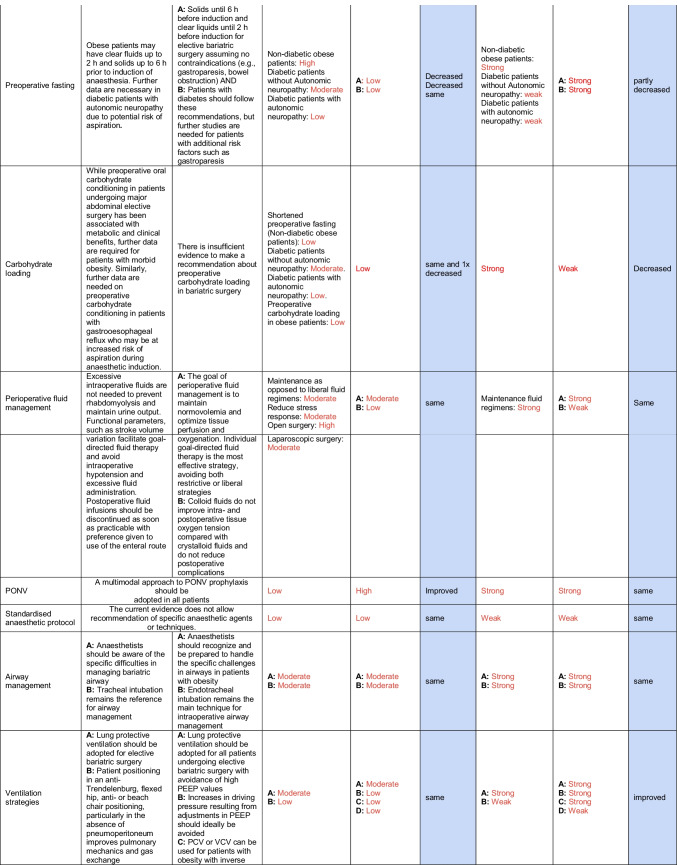

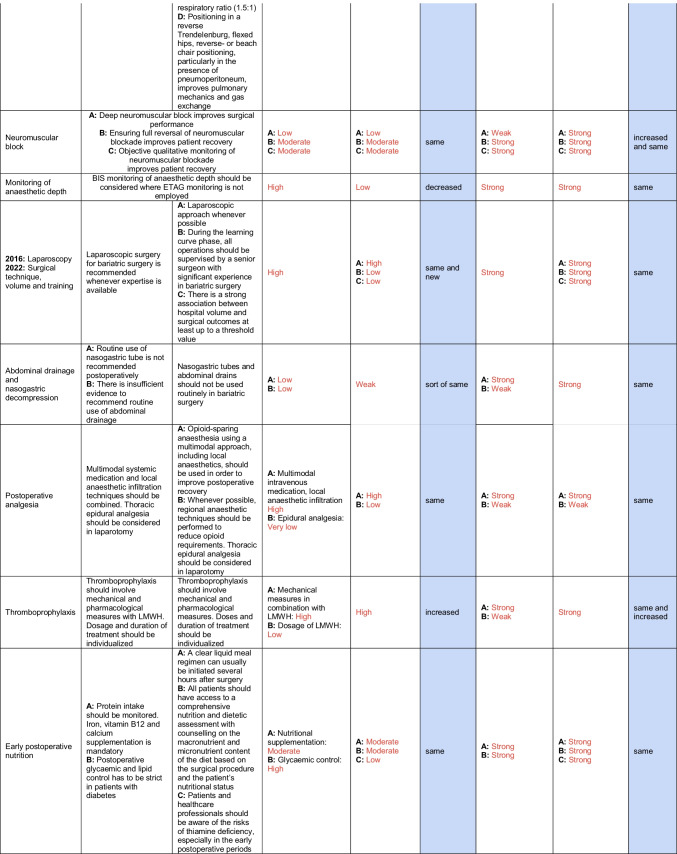

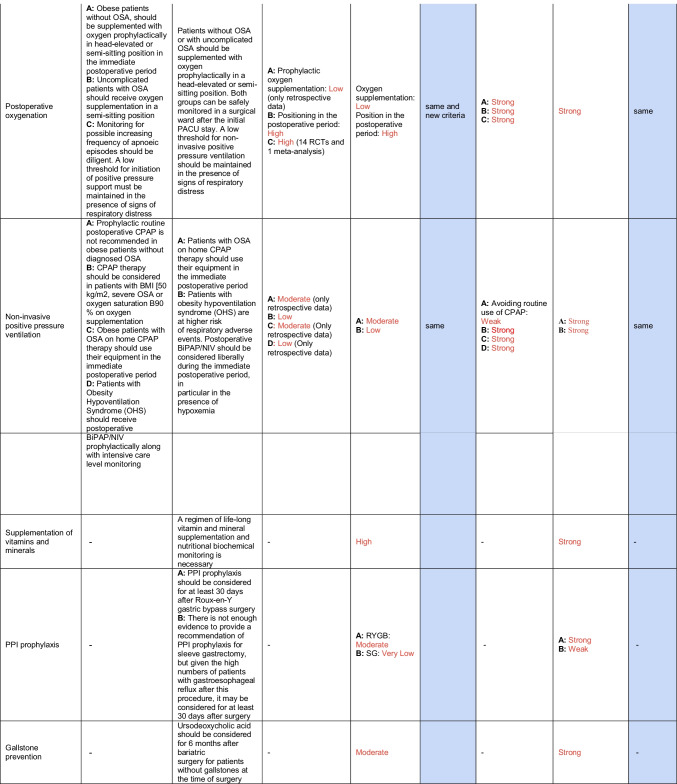
ERABS comparisons between the guidelines from 2016 and 2022

Among the recommendations in G16, ten of them were classified as weak and 26 of them as strong. In G22, there were eight weak recommendations and 32 strong recommendations.

### *S*trong Recommendations with a Low LoE (Table 1)

This was noted nine times in G16 and fourteen times in G22.

(Preoperative consulting G22; alcohol G16, G22; postoperative weight loss G16, G22; diabetes G22; glucocorticoids G16; preoperative fasting (2 sub-elements) G22; carbohydrate loading G16; PONV G16; ventilation strategy (2 sub-elements) G22; deep neuromuscular block G22; nasogastric tube G16; early postoperative nutrition G22; prophylactic oxygen G16 and G22; non-invasive positive pressure G16/G22; surgical technique G16 (2 sub-elements) and G22).

### Strong Recommendations with a Moderate LoE (Table 1)

This was noted thirteen times in G16 and fourteen times in G22.

(Preoperative consulting G16; indications/contraindications G22, smoking G22, preoperative weight loss/postoperative complications G22; DM autonomic neuropathy G16; carbohydrate loading G16; preoperative fluid management G16 (3 sub-elements) and G22; airway management (2 sub-elements) in G16 and G22; ventilation strategy/lung-protective ventilation G16 and G22; reversal neuromuscular block and monitoring (2 sub-elements) in G16 and G22; early postoperative nutrition/dietetic assessment G16 and (2 sub-elements) G22; non-invasive OSA and CPAP G16 and G22; PPI RYGB G22; gallstone prevention G22).

### Strong Recommendations with a High LoE (Table 1)

This was noted eleven times in G16 and six times in G22.

(Smoking G16; post-operative complications G16; preoperative fasting G16; perioperative fluid management/open surgery G16; PONV G22; monitoring anesthesia depth (BIS) G16; surgical technique/laparoscopic G16 and G22; postoperative analgesia management G16 and G22; thromboprophylaxis/LMWH G16 and G22; early postoperative nutrition/glycemic control G16; postoperative oxygenation by position and monitoring apnea G16 (2 sub-elements) and G22; supplementation of vitamins and minerals G22).

### Weak Recommendations with a Low LoE (Table 1)

This was noted eight times in G16 and eight times in G22.

(Prehabilitation and exercise G16 and G22; glucocorticoids G22; preoperative fasting G16; carbohydrate loading G22; perioperative fluid management G22; standardized anesthetic protocol G16 and G22; ventilation strategies G16 and G22; neuromuscular block G16; abdominal drainage and nasogastric decompression G16; postoperative analgesia G16 and G22; thromboprophylaxis G16; PPI prophylaxis sleeve G22).

### Weak Recommendations with a Moderate LoE (Table 1)

This was noted twice in G16 and 0 times in G22.

(Preoperative fasting G16; non-invasive positive pressure ventilation G16).

### Weak Recommendations with a High LoE (Table 1)

This was noted 0 times in G16 and 0 times in G22.

### Changes/Improvements in LoE of Elements Between 2016 and 2022 (Table 1)

Low LoE in G16 that remained low in G22 was noted for 13 elements.

(Prehabilitation and exercise, smoking and alcohol cessation, preoperative weight loss, glucocorticoids, preoperative fasting, carbohydrate loading, standardized anesthetic protocol, ventilation strategies, neuromuscular block, abdominal drainage and nasogastric decompression, postoperative analgesia, postoperative oxygenation, non-invasive positive pressure ventilation).

Low LoE in G16 that increased to moderate LoE in G22 was noted once.

(Non-invasive positive pressure ventilation element).

Low LoE in G16 that changed to high LoE in G22 was noted for two elements.

(PONV and thromboprophylaxis).

Moderate LoE in G16 that reduced to low LoE in G22 was noted for six elements.

(Preoperative information, education, and counselling, preoperative fasting, carbohydrate loading, perioperative fluid management, ventilation strategies, non-invasive positive pressure ventilation).

Moderate LoE in G16 that remained moderate in G22 was noted for six elements.

(Perioperative fluid management, airway management, ventilation strategies, neuromuscular block, early postoperative nutrition, non-invasive positive pressure ventilation).

Moderate LoE in G16 that increased to high LoE in G22 and high LoE in G16 that reduced to low LoE in G22 were noted 0 times.

High LoE in G16 that reduced to moderate LoE in G22 was noted once.

(Preoperative weight loss).

High LoE in G16 that remained high in G22 was noted for four elements.

(Laparoscopy/surgical technique, postoperative analgesia, thromboprophylaxis, postoperative oxygenation).

## Discussion

This review analyzed two guidelines to see how they scored their results across all elements over both periods and what improved or worsened between the two publication periods. This review also investigated how the risk of bias (RoB) was presented or extracted and how this involved the LoE.

Between the two guidelines publication periods, the extracted research papers still showed 13 times LoE as low, and therefore, there was no improvement in research quality or lowering of bias. In six instances, a moderate LoE decreased to lower LoE in G22. Four elements had high LoE that remained so; the same was applicable for six elements with moderate LoE.

After analyzing all the results, this review created new possible recommendations for the future. This will be the discussion of a Global Consortium of Bariatric Care and Research, points of interest towards improving RoB and FAIR, and metadata incorporation for lowering BIAS and increasing research quality.

This review looked at the GRADE assessment for RoB. This RoB has five topics: selection bias, detection bias, attrition bias, reporting bias, and confounding bias. This generally will be scored as − (low RoB), + (high RoB), and ? (unclear RoB). The LoE in GRADE has four categories, high, moderate, low, or very low. In summary, the LoE relies on how the RoB is scored. Therefore, every study must evaluate and highlight the LoE and how this conclusion is drawn to know if a study has a “good” or “bad” methodology background.

Both G16 and G22 present LoE and recommendations. Both guidelines did not explain how the LoE conclusions were reached regarding RoB with all the mentioned chapters above. There was no discussion on how requests with low LoE can be avoided in future studies or if this was important.

A possible explanation can be that this was never the purpose of both guidelines to present all the in-depth improvements in methodology in research. Therefore, this review highlighted this aspect to move forward with a new chapter on methodology improvements in bariatric care research.

The solution should be that all presented research papers get a new assessment on the RoB topics and summarize how this new assessment can affect the results of the individual studies and, therefore, the followed recommendations. Furthermore, this review and evaluation of all the results can help other researchers conduct further research to understand where, how, and what can be changed to increase the level of evidence and lower any possible risk of bias. Still, 13 LoE stayed low between both guidelines’ searches.

### GCBCR

There should be an opportunity for IFSO and all the local societies to collaborate and therefore create a team of experts that comes together in creating a framework in the perhaps possible to mention Global Consortium of Bariatric Care and Research (GCBCR) Network (or any suitably named organization). This is necessary because it is essential to gain insight into how the G16 and G22 established their LoE. Which of the five elements of the RoB was the “problem,” and what can and could be done in the future? For now, this question stays unanswered, and this was the central research question of this review. A framework can help every researcher increase methodology and outcome in research for ERABS guidelines and use this information in all new studies as a new fundament. Since not all biases can be solved and are always present in a study, a clear overview and solution in a framework can help answer these raised questions.

Also, we want to acknowledge both teams in conducting the guidelines [4, 5]. The guidelines significantly improved care, lowering the duration of hospitalization and reducing complications [11–16]. The reason why scientists conduct research is to remain critical and evaluate where improvements can be made. In both G16 and G22, there was some lack of description of how studies were assigned low or high LoE; also, how a strong recommendation with a low LoE was justified.

### Points of Interest Towards Improving RoB

Since bias is the main unanswered question in the results from both G16 and G22 in the quality of research, it is necessary to formulate solutions to prevent this in the future. Therefore, within the GCBCR, a new framework with five improvements or recommendations for lowering RoB and increasing the LoE should be performed and discussed: With a little impetus, (1) selection bias, for how random sequence generation, and allocation concealment, if occurring if randomization is not possible, can be corrected with good fundaments in knowing what statistical tests can be used, in combination with the distinction between selection and information bias in conducting a study; (2) detection bias, in how good selection and distinguishing between selection and information bias can help increase the quality [18–21]; (3) attrition bias, in how “low-hanging fruit” can be tackled relatively easily and can increase the study quality fast, is the different rates of losses to follow-up in the exposure groups, which may change the characteristics of these groups irrespective of the studied intervention [22, 23]; (4) reporting bias on how selective reporting threatens the validity of the published data if the decision to report depends on the nature of the results and how this can be resolved. [24–26]; and last to discuss is how (5) confounding bias can arise from completely unmeasured confounders with errors specific to observational research, common in the bariatric trials performed by Ciocănea-Teodorescu and Sjölander [17].

#### FAIR

As mentioned in the solutions toward a new framework within the GCBCR, the introduction of FAIR data (findable, accessible, interoperable, and reusable data) and metadata must be considered as the new standard in future research for an excellent basic fundament, better level of evidence, the possibility for data sharing, and a better understanding of how the risk of bias is constantly reacting between and within studies.

In 2021, Springer Nature [27] published a white paper whereby the evaluation of 5-year FAIR data was described and looked at the real-world impact of FAIR and the considerations of what will be next for research data and open science. In March 2016, the FAIR was introduced [28], and the G20 leaders endorsed the FAIR principles in future research. [29]. In 2018, the European Commission published its report and action plan to turn FAIR into reality [30]. But at the same time, a survey in 2020 that asked nearly 5000 researchers in over 190 countries showed that 39.4% of survey respondents had never heard of the FAIR principles before taking the survey, and 36.2% had heard of the FAIR principles but were not familiar with them, compared with only 24.4% who are familiar with the FAIR principles. From another field, the pandemic has made a case for data sharing and increased the need for FAIR. As the virus spread in early 2020, many governments and their research funding agencies had a significant and rapid response. One novel addition to COVID-19 funding opportunities was the adoption of both FAIR and open data principles [31].

Data and the possibilities of increasing quality and working together are now known to governments. However, they are not yet sufficiently for scientists.

In this context, to better understand the effect of FAIR data, FAIR has four critical elements to improve research infrastructure, making it easier for researchers to collaborate, ultimately improving healthcare quality. A quick look at the four elements shows us findable — data and metadata that humans and computer systems should quickly locate; accessible — what is stored long term, so that they can easily be accessed and downloaded with well-defined licenses and access conditions; interoperable — ready to be combined with other datasets by humans or computers; and reusable — ready to be used for future research and further processed using computational methods [32–35].

### Barriers and Limitations

A point of emphasis for the GCBCR and FAIR data is how to implement this global research consortium with the ownership of data, privacy concerns, and variations in how countries treat such data requests etcetera.

Start addressing that not only the bariatric surgeon (at the end responsible for the patient) is the main stakeholder within ERABS guidelines. This is a truly multi-disciplinary approach. Because every discipline dietician, psychologist, anesthesiologist, etc., within the GCBCR should be a part of this discipline, sub-teams should therefore be created to lower the workload and possibilities to have a clear outcome within all the elements. Therefore, good communication between all the sub-teams is challenging but necessary.

A limitation within this project could be the factors of inter-cultural and inter-continental differences, respectively. Examples are that medication is not the same or present, protocols are possible not be approached the same everywhere, and medical ethical boards within every country or hospital could have different opinions on how “good” research should be approached or carried out. Therefore, a good alignment is the main goal for a better level of evidence, and therefore the inter-cultural and inter-continent differences should also be addressed when a GCBCR is created and not only focus on the elements from a guideline. A possible solution to have a good overview is that all elements can be scored as a “traffic light” approach (green, orange, red) low-hanging fruit and easy implementation as green to very difficult because of all the points mentioned above as a GCBCR bias. In the end, barriers and limitations are something that can be resolved if we want to increase the level of evidence of all the studies and get a good understanding of how the RoB is constructed in every element.

## Conclusion

The 2016 and 2022 editions of the ERABS guidelines reveal that there are still significant qualitative improvements to be made in the LoE and an RoB concerning the recommendations provided. It would be advantageous for the bariatric surgical community to establish a global research consortium with all the multi-disciplinary stakeholders combined and working together. Along with creating an exemplary data storage system that is FAIR compliant and more attention to statistical and methodological implementation, better guidelines and improved patient care are achievable in the future.
